# A peer support program results in greater health benefits for peer leaders than other participants: evidence from the Kerala diabetes prevention program

**DOI:** 10.1186/s12889-023-16049-0

**Published:** 2023-06-19

**Authors:** Tilahun Haregu, Zahra Aziz, Yingting Cao, Thirunavukkarasu Sathish, Kavumpurathu Raman Thankappan, Jeemon Panniyammakal, Pilvikki Absetz, Elezebeth Mathews, Sajitha Balachandran, Edwin B. Fisher, Brian Oldenburg

**Affiliations:** 1 Baker Heart and Diabetes Institute, Melbourne, Australia; 2 Nossal Institute for Global Health, University of Melbourne, Melbourne, Australia; 3 School of Psychology and Public Health, La Trobe University, Melbourne, Australia; 4 School of Public Health & Preventive Medicine, Monash University, Melbourne, Australia; 5 Department of Sport, Exercise and Nutrition Sciences, School of Allied Health, Human Services and Sport, La Trobe University, Melbourne, Australia; 6 Department of Family and Preventive Medicine, Emory University, Atlanta, GA USA; 7 Amrita Institute of Medical Sciences, Kochi, Kerala India; 8 Achutha Menon Centre for Health Science Studies, Sree Chitra Tirunal Institute of Medical Science and Technology, Kerala, India; 9 Faculty of Social Sciences, Tampere University, Tampere, Finland; 10 Department of Public Health and Community Medicine, Central University of Kerala, Kasaragod, Kerala India; 11 Population Research Centre, University of Kerala, Trivandrum, Kerala India; 12 Gillings School of Global Public Health, University of North Carolina, Chapel Hill, USA

**Keywords:** Peer support, Diabetes, Cardiometabolic risk, Kerala, India

## Abstract

**Background:**

Peer support programs are promising approaches to diabetes prevention. However, there is still limited evidence on the health benefits of peer support programs for lay peer leaders.

**Purpose:**

To examine whether a peer support program designed for diabetes prevention resulted in greater improvements in health behaviors and outcomes for peer leaders as compared to other participants.

**Methods:**

51 lay peer leaders and 437 participants from the Kerala Diabetes Prevention Program were included. Data were collected at baseline, 12 months, and 24 months. We compared behavioral, clinical, biochemical, and health-related quality of life parameters between peer leaders and their peers at the three time-points.

**Results:**

After 12 months, peer leaders showed significant improvements in leisure time physical activity (+ 17.7% vs. + 3.4%, P = 0.001) and health-related quality of life (0.0 vs. + 0.1, P = 0.004); and a significant reduction in alcohol use (-13.6% vs. -6.6%, P = 0.012) and 2-hour plasma glucose (-4.1 vs. + 9.9, P = 0.006), as compared to participants. After 24 months, relative to baseline, peer leaders had significant improvements in fruit and vegetable intake (+ 34.5% vs. + 26.5%, P = 0.017) and leisure time physical activity (+ 7.9% vs. -0.9%, P = 0.009); and a greater reduction in alcohol use (-13.6% vs. -4.9%, P = 0.008), and waist-to-hip ratio (-0.04 vs. -0.02, P = 0.014), as compared to participants. However, only the changes in fruit and vegetable intake and waist-to-hip ratio were maintained between 12 and 24 months.

**Conclusion:**

Being a peer leader in a diabetes prevention program was associated with greater health benefits during and after the intervention period. Further studies are needed to examine the long-term sustainability of these benefits.

## Introduction

Peer support is an established intervention in which peers offer support to others with whom they share important circumstances or characteristics [1]. In addition to sharing a common health problem or condition [2], peers may also live in the same community as well as also sharing similar social and economic challenges [3, 4]. Peer support may include the provision of psychosocial support, information support, and practical assistance that can help reduce health risks, increase adherence to treatment, and/or improve quality of life [5]. Traditionally, peer supporters/leaders are community residents (including community health workers, lay health advisers, a family member, or a friend) with a similar lived experience and are willing to provide their peers with various types of support. Peer supporters/leaders are not generally required to have specialized knowledge, nor to be medical experts or health professionals [6].

An important and emerging area for peer support is the increasing number of people at high risk of developing diabetes in low- and middle-income countries (LMICs). This increase is due to rapid economic growth, urbanization, nutrition transition and changes in lifestyle factors. Subsequently, the need for effective diabetes prevention programs is increasing [7]. In India, the number of people with diabetes is estimated to be the second highest in the world [8]. Given the lack of human resources for health, this causes more health system challenges in terms of improving access to and quality of health services for people with diabetes.

Peer support programs are considered as promising approaches for improving complex health behaviours, such as healthy diet and physical activity, in both diabetes prevention and management [9, 10]. Peer support is an effective strategy for reaching people with complex health behaviors in diverse settings that health services too often fail to engage. Peer support can also play a critical role in mitigating common diabetes risk factors and reducing the risk of developing diabetes among high-risk individuals [10]. Additionally, there is a large body of evidence supporting the positive impact of peer support programs on the improvement of self-management of diabetes and adoption of healthy behaviors by increasing knowledge and feeling of social connectedness that can lead to better health outcomes [11]. The peer support approach has been considered to be feasible, cost-effective, and flexible for improving diabetes care and outcomes and reducing the risk of complications [12].

Most research on peer support interventions was conducted in high-income countries. A review that examined Peer Support for complex, sustained health behaviors in prevention or disease management with emphasis on diabetes prevention and management[13] identified 65 studies of which 53 were from World Bank-designated high-income countries and 12 from low-income and middle-income countries. Studies in high-income countries have also focused on the benefits of such interventions for “safety net” healthcare settings or groups subject to racial, social, or economic injustice[14–16]. Evidence from such studies demonstrated the potential of peer support programs in improving health behavior and outcomes among people at high-risk for diabetes.

Although there is an enormous amount of research showing the value of peer support and related approaches, a question often raised concerns the effect on the peer leaders themselves. Very few studies have evaluated the impacts on lay peer leaders. Among these is a study that reported the benefits of peer support programs for peer leaders in terms of improvements in interpersonal, communication, and leadership skills [17]. It has also been indicated that peer support programs enable peer leaders to establish stronger linkages with health services and encourage their peers to seek medical support on a timely basis [18]. A study among peer supporters for diabetes management in Hong Kong [19] found that people with diabetes who were involved in providing peer support to other people with diabetes had improved their self-care while maintaining glycaemic control over four years period.

Further evidence on the impacts of peer support interventions on health behaviors and outcomes of peer leaders could inform the design, adaptation and scale-up of such programs. Evidence of the health benefits of being a peer leader/supporter could encourage more people to volunteer as peer leaders. Such evidence will also inform the quality improvement of peer support programs. In this study, we report on the impacts of peer support interventions among peer supporters engaged in diabetes prevention in India. Our study examined the health benefits of being a peer leader in a group-based, peer-led diabetes prevention program in Southern India, the Kerala Diabetes Prevention Program (K-DPP), on peer leaders’ health behaviors and health outcomes.

## Methods

### Kerala diabetes prevention program

The original K-DPP trial was a cluster-randomized trial of a group-based and peer-led diabetes prevention intervention implemented in the State of Kerala, India [20]. The methodology of K-DPP has been reported in detail elsewhere [20–23]. In brief, 60 polling areas (geographically designated electoral divisions) were randomly assigned to a peer support intervention group (30 polling areas or clusters) or a control group (30 polling areas). The study recruited 500 intervention and 507 control participants aged between 30 and 60 years and at high risk of diabetes based on the Indian Diabetes Risk Score (IDRS) of ≥ 60 and without diabetes based on a 2-hour oral glucose tolerance test [24].

The 12-month peer support program comprised 15 interactive group sessions based on a standardized curriculum with a peer leader handbook that supported the activities and provided information; and detailed instructions and activities for each session [22, 25]. The curriculum was adapted from United States Diabetes Prevention Program[26], Finnish Good Ageing in Lahti Region (GOAL) program [27] and the Australian Greater Green Triangle (GGT) Diabetes Prevention Project [28] through situational analysis, needs assessment, and cultural translation [22, 29]. Each session lasted for 60–90 min. The 15 sessions included an introductory session delivered by the K-DPP team, two education sessions conducted by local experts and 12 monthly sessions delivered by trained peer leaders [22, 23]. Topics covered during the group sessions included diabetes prevention, improving physical activity, fruit and vegetable intake, reduce salt and sugar, reduce calories to maintain a healthy weight, sleep improvement, reduce alcohol use, and tobacco cessation. Peer group activities included setting and monitoring of individual goals for physical activity, diet, smoking and alcohol use. Goal setting as an intervention activity included only behavioral goals. Goal setting and planning was guided but individualised – i.e., participants selected their goals based on the self-monitoring they had conducted. In addition to individual goals, the groups were encouraged to set group goals (e.g., some groups set goals for walking together, and cleared walking paths for this purpose). Based on the cultural adaptation and needs assessment, the Participant Workbook included tools for self-monitoring of lifestyle factors; setting individual goals for lifestyle factors (diet, physical activity, tobacco and alcohol use); and regular review of attainment of their goals [22, 30]. Intervention participants used goal setting, action planning and self-recording of activities in their workbook as key behavioral strategies for improving physical activity and healthy eating [21]. Peer leaders and LRPs encouraged and supported participants in their attainment of their goals by organizing peer group activities and community engagement activities. The control group received a health education booklet at baseline. Data were collected at baseline, 12 months, and 24 months. Additionally, regular monitoring of the delivery of the intervention generated additional data on process indicators.

After 24 months of follow-up, 17.1% of control participants and 14.9% of intervention participants developed diabetes. At 24 months, intervention participants had a greater reduction in IDRS as compared to control participants. Additionally, intervention participants had a higher reduction in alcohol use and a significant increase in fruit and vegetable consumption. They also had significantly higher physical functioning scores of the health-related quality of life (HRQoL) scale as compared to control participants [21]. Most important, perhaps, was a replication of the key finding of major, international studies of diabetes prevention such as in Finland [31] and the US [26] showing significantly reduced rates of conversion to diabetes among those with impaired glucose tolerance at baseline.

### Selection of peer leaders

Peer leaders were selected from among the group members by the members themselves. This was to reinforce the social connections among peer leaders and group members and the identification of the peer leaders with the program. There were 30 peer support groups with 10–23 participants each. During the first group session, the K-DPP team clearly explained the responsibilities of peer leaders to each group. The participants were then asked to discuss among themselves and nominate two potential candidates from their group for the role of peer leader. Each group identified and nominated two peer leaders (one male, one female) based on their social credibility, willingness to lead the group, and acceptability to other group members.

The responsibilities of peer leaders included participating in the peer leaders’ training (led by the K-DPP team), facilitating the peer group sessions with the support of the intervention team, and maintaining contact and communication with group participants outside group meetings to provide them with additional informational and emotional support. In addition, their roles included developing a good rapport with the group members organizing the venue for group sessions, assisting in goal setting for the group participants organizing community-based activities with the support of Local Resource Persons (LRPs) identified by the K-DPP and preparing group session reports.

### Training and mentoring of peer leaders

The K-DPP training team comprised of an intervention manager (registered nurse with public health training) and an intervention assistant (medical social worker), delivered two peer leader training sessions. The purpose of the training was to build peer leaders’ knowledge about diabetes and enhance their skills in leading peer groups and facilitating peer group sessions. Peer leaders were trained on how to enable group members in setting goals, and monitoring progress towards goals; provide informational and emotional support to group members; and organizing community engagement activities. The training also aimed to improve peer leaders’ skills in group facilitation and communication. In total, out of 60 peer leaders selected (two for each group – one male and one female), 51 attended the peer leader training sessions. Those who did not attend the training were not retained as peer leaders. This resulted in all groups having at least one trained peer leader over the duration of the intervention.

A two-day refresher training was provided after the eighth peer group session. Other support provided to peer leaders during the program included support from LRPs, mostly community health workers (also called Accredited Social Health Activists). Peers were also trained to help community members access health services. Peer leaders also received ongoing support, including encouragement to effectively lead peer support groups throughout the program and addressing any queries they might have, from K-DPP team throughout the intervention period, and two peer leaders’ forums were conducted to facilitate knowledge exchange and sharing of learnings among peer leaders.

Peer leaders set their goals and work toward the achievement of their goals in the same way and on the same timeline as the rest of the intervention group participants. The goal setting and monitoring processes were the same between peer leaders and other participants. However, peer leaders, together with LRPs, had an additional role of assisting other participants set their goals and work towards the achievement of their goals.LRPs were selected from among community leaders or other influential citizens. They provided practical support to peer leaders in organizing the logistics for local program delivery. Wherever possible, the LRPs also attended group sessions as observers for their respective communities and completed Observers’ Checklists for follow-up of the progress of group sessions. They completed observer’s checklist for the first peer-led session of 28 of the 30 peer groups and 62% of all the remaining subsequent peer-led group sessions, i.e. sessions 2–15.

### Data source, variables, and measurement

K-DPP participants completed baseline, 12-month, and 24-month assessments. The details of the study methods used in the K-DPP study have been reported elsewhere [20]. In this analysis, we used data from training assessment, monitoring of delivery of 12 peer group sessions conducted over the 12 months and the assessments at the three time-points for the intervention group only.

We included the following categories of variables in this study:

1. *Sociodemographic characteristics* of peer leaders and their peers at baseline including gender, age, marital status, education, occupation, and estimated annual household expenditure for the previous year.
2. *Behavioural characteristics* included self-report measures of diet (number of servings of fruit and vegetables on a typical day); participation in leisure time physical activity (assessed using self-reported moderate physical activities during leisure time in bouts of at least 10 min duration and vigorous physical activities during leisure time in bouts of at least 10 min duration [Flash cards were used to describe these to participants]. Participants with at least one of these were considered “Yes” for their participation in leisre time physical activity); alcohol consumption in the past 30 days; and current smoking.
3. *Clinical characteristics* of participants included body weight (Kilograms), waist circumference (centimeters), waist-to-hip ratio, blood pressure, and Indian Diabetes Risk Score (IDRS) score.
4. *Biochemical characteristics* included blood glucose measures (fasting plasma glucose, 2-hour plasma glucose, Glycated hemoglobin (HbA1c), lipid measures (LDL)), and conversion to diabetes among those with Impaired Glucose Tolerance at baseline. Diabetes was diagnosed according to the American Diabetes Association criteria (FPG ≥ 7.0 mmol/l and/or 2-hr PG ≥ 11.1 mmol/l).
5. HRQoL was measured by Short Form 36. We used the physical component summary, mental component summary, and SF-6D in our analysis. Most of these variables were assessed at baseline, 12 months, and 24 months. Details of the measurement of these variables have been published [21].

### Data analysis

We used Stata 16.0 for data analysis. We described the characteristics of peer leaders and peers at baseline, 12 months, and 24 months using proportions, mean (standard deviation,) and median (interquartile range). We tested the differences in these characteristics between the two groups at baseline, 12 months, and 24 months using chi-square test, t-test or Wilcoxon signed rank test, as appropriate.

Peer leaders, as leaders of the group, were also members of peer group. However, in this analysis, peer leaders and other participants were mutually exclusive. In our analysis, we included peer leaders and participants who completed all three rounds of data collection (baseline, 12 months, and 24 months). We conducted analysis of the health benefit of being a peer leader in three steps:

#### Effects at 12 months (peer leaders)

Analysis of differences among peer leaders between baseline and 12 months.

#### Effects at 12 months (peer leaders vs. participants)

Analysis of differences between peer leaders and peers at 12 months controlling for baseline values.

#### Effects at 24 months (peer leaders vs. participants)

Analysis of differences between peer leaders and peers at 24 months controlling for baseline values.

To assess effects at 12 months among peer leaders, we used McNemar’s Chi-square test for categorical variables and paired t-test for continuous variables. To assess effects at 12 months and 24 months between peer leaders and participants, we used logistic regression-based pairwise comparison to compute differences in predicted proportions; and ANOVA with multiple comparison to compute differences in marginal means. These models were adjusted for age, gender and baseline values of the respective outcome variables to account for possible effects of age, gender and baseline status of the outcome variables on the differences in the outcome variables at 12 and 24 months. These covariates were selected based on evidence of their association with the outcome variables. P values less than 0.05 were considered to be statistically significant.

## Results

### Characteristics of peer leaders and other participants

Of the 60 peer leaders (two per group; one male and one female) who were identified at the beginning of the program, nine (four males and five females) dropped out due to reasons including relocation for work, caregiver responsibility at home and time constraint due to other commitments. Fifty-one peer leaders (85%) attended the 2-day peer leader training of whom 49 continued as peer leaders over the entire duration of the intervention. Forty-eight peer leaders attended the 2-day refresher training which was provided after the eighth peer group session. Fifty-one peer leaders were interviewed at 24 months. Of the 49, 24 were women with mean age of 42.3 years (SD = 6.1) and 25 were men with mean age of 46.8 (9.0).

Most of the peer leaders were married (94%), employed (76%) and educated up to secondary school or above (63%). Those who were in paid employment, worked on an average of 32.6 (SD + 18.3) hours per week. Most of the peer leaders had not used any tobacco products (90%) or not consumed alcohol (78%) in the previous 30 days. Females peer leaders were younger (42.3 vs. 46.8 years), less highly educated (16% vs. 38% tertiary education), tobacco non-users (0% vs. 19%) and working lower number of hours per week (26 vs. 37 h), compared to male peer leaders. Out of the 500 participants enrolled in the intervention group, altogether 437 participants and 51 peer leaders were interviewed at all the three time points of assessment. Table 1 shows sociodemographic characteristics of the peer leaders and participants.

**Table 1:** Sociodemographic characteristics of peer leaders and participants

	Participants (n = 437)	Peer leaders (n = 51)
Female, n (%)	212(48.5)	25(49.0)
Age(years), mean (SD)	46.3(7.5)	44.6(7.9)
Education, n (%)		
Up to primary	131(30)	0(0)
Middle	110(25.2)	14(27.5)
Secondary and above	166(44.8)	37(72.5)
Occupation, n (%)		
Skilled/unskilled	317(72.5)	39(76.5)
Homemaker	117(26.8)	11(21.6)
Unemployed/retired	3(0.7)	1
Monthly household expenditure in INR, median (IQR)	7000(5000 to 10,000)	7500(5000 to 10,000)
Marital status		
Married	417(95.4)	48(94.1)
Others	20(4.6)	3(5.9)

### Attendance of peer group sessions

Peer leaders and participants differed in the number of sessions they attended. Peer leaders attended almost all sessions. The median number of peer group sessions attended by peer leaders was 14 (Interquartile range: 12,15). The median number of sessions attended by other participants was 9 (Interquartile range: 3,13).

### Comparison of health behaviours and outcomes at baseline

As indicated in Table 2, participants and peer leaders differed on a number of variables at baseline. Peer leaders were more likely to report ≥ 5 servings of fruits or vegetables per day, less likely to report tobacco us and reported greater health related quality of life. On clinical and biochemical measures, they had notably lower systolic blood pressure (116.7 vs. 125.7 mmHg) and LDL cholesterol (142.7 vs. 155.4 mg/dl). Although chosen from among participants, these as well as the difference in education (72.5% secondary and above vs. 44.8 among other participants) suggests that the groups chose those who, while still at risk, were nevertheless better educated and healthier.

**Table 2:** Behavioural, clinical, and biomedical characteristics of participants across three time points (bivariate analysis)

	Baseline			12 months			24 months		
	**Participants**	**Peer leaders**	**P**	**Participants**	**Peer leaders**	**P**	**Participants**	**Peer leaders**	**P**
Behavioural characteristics									
>=5 servings of fruit & veg./day, n (%)	127(29.1)	22(43.1)	0.039	254(58.1)	29(56.7)	0.863	243(55.6)	38(74.5)	0.010
Physically active, n (%)	86(19.7)	15(29.4)	0.105	101(23.1)	24(47.1)	0.000	82(18.8)	19(37.3)	0.002
Alcohol use, n (%)	99(22.7)	11(21.6)	0.861	79(18.1)	4(8.0)	0.073	76(17.8)	4(8)	0.079
Current smoking, n (%)	94(21.5)	5(9.8)	0.049	70(16)	3(6)	0.060	66(15.5)	4(8)	0.159
Clinical characteristics									
Weight (Kg), mean (SD)	62.4(11.6)	64.1(12.1)	0.327	62.9(11.8)	63.7(11.9)	0.649	63.5(11.9)	65.1(12.4)	0.399
Waist circumference (cm), mean (SD)	87.9(9.8)	87.9(9.5)	0.999	86.5(10.7)	83.7(10.3)	0.082	88.1(10.1)	86.3(9.6)	0.231
Waist-to-hip ratio, mean (SD)	0.933(0.1)	0.934(0.1)	0.942	0.911(0.1)	0.884(0.1)	0.037	0.916(0.1)	0.894(0.1)	0.025
Systolic BP (mmHg), mean (SD)	125.7(18.3)	116.7(14.3)	0.001	124.1(18.7)	114.9(13.3)	0.001	123.8(18.5)	116.7(14.2)	0.010
IDRS Score, mean (SD)	67.1(8.4)	65.3(7.3)	0.145	60.3(12.7)	55.8(12.8)	0.145	61.4(12.9)	59.2(14)	0.261
Biochemical characteristics									
FPG (mg/dl), mean (SD)	104.2(9.1)	101.9(10.1)	0.089	107.2(11.6)	103.1(9.9)	0.018	108.4(15.4)	106.7(15.3)	0.474
2-hr plasma glucose(mg/dl), mean (SD)	106.6(28.5)	103.8(28.9)	0.507	116.5(36.7)	99.7(29.3)	0.002	112.1(34.7)	105.2(44.5)	0.219
HbA1c (percent), mean (SD)	5.59(0.47)	5.53(0.53)	0.362	5.58(0.46)	5.45(0.52)	0.078	5.61(0.53)	5.44(0.49)	0.038
LDL cholesterol (mg/dl), mean (SD)	155.4(35.4)	142.7(27.8)	0.014	155.4(34.3)	141.1(34.2)	0.006	150.4(33.8)	137.8(29.2)	0.013
Health-related quality of life*									
Physical component sum., mean(SD)3	48.9(8.8)	51.7(6.8)	0.025	51.6(8.2)	54.9(5.2)	0.005	50.3(8.1)	52.2(7.7)	0.121
Mental component sum., mean(SD)	53.8(9.2)	55.6(8.3)	0.182	56.1(7.9)	58.3(6.3)	0.065	56.2(7.6)	58.8(5.9)	0.020
SF-6D, mean (SD)	0.8(0.2)	0.8(0.1)	0.119	0.8(0.2)	0.9(0.1)	0.004	0.8(0.1)	0.9(0.1)	0.037

*Higher scores show better quality of life; FPG = Fasting Plasma Glucose; PL = Peer Leader.

At all time points peer leaders (n = 51); and participants (n = 437). P values < 0.05 were considered to be statistically significant.

### Changes in health outcomes among peer leaders

As detailed in Table 3, peer leaders had a significant reduction in alcohol use (-14%) at 12 months, compared to baseline. Average waist circumference and WHR among peer leaders decreased by 4.1 cm and 0.05, respectively, between baseline and 12 months. Similary, there was a statistically significant reduction in average IDRS scores among peer leaders between baseline and 12 months. Moreover, a significant improvement in HRQoL measures were observed at 12 months. Inspection of Table 3 shows that peer leaders maintained the decreased use of alcohol, improvements in WHR and mental component of HRQoL and SF-6D at 24 months. However, the changes for waist circumference, IDRS and physical quality of life were only partially sustained.

### Changes in health outcomes among other participants

Other participants showed significant improvements from baseline to 12 months on fruit and vegetable intake, alcohol consumption, tobacco use, WHR, blood pressure, and HRQoL and maintained those changes for all these measures at 24 months. Note that tests of these within group changes were considerably more sensitive than those among peer leaders because of the differences in sizes of the two samples.

### Comparison of changes between peer leaders and other participants

Table 3 includes comparisons of groups at 12 and 24 months controlling for baseline levels of the same variables so that the 12- and 24-month differences reflect differences in changes from baseline to those two assessments. As also described in Table 3, peer leaders showed significantly greater improvements in participation in leisure time physical activity, alcohol use, body weight, and 2-hour plasma glucose levels, physical components summary and SF-6D of HRQoL. At 24 months, peer leaders showed significantly greater changes from baseline for ≥ 5 servings of fruit and vegetable per day, physical activity, alcohol use, waist circumference, and waist-to-hip ratio. Summarizing these differences, peer leaders showed greater changes from baseline to 12 months on 6 of 16 variables and greater overall changes from baseline to 24 months also on 5 of 16 variables.

### Maintenance of changes from 12 to 24 months

Differences in sustaining changes from 12 to 24 months were assessed by comparing peer leaders and other participants on 24-month values controlling for both baseline and 12-month values. In these analyses, changes in fruit and vegetable intake and WHR were statistically significant indicating greater maintenance of these changes among the peer leaders.

**Table 3:** Comparison of behavioural, clinical, and biomedical characteristics of peer leaders and participants (Multivariate analysis)

	Effect at 12 months (PLs) (n = 51)		Effect at 12 months – PL (n = 51) vs. Participants (n = 437)		Overall effect at 24 months – PL (n = 51) vs. Participants (n = 437)	
	**Difference (95% CI)**	**P**	**Difference (95% CI)**	**P**	**Difference (95% CI)**	**P**
Behavioural characteristics [Proportions]						
>=5 servings of fruit & vegetable/day	0.14(-0.06,0.34)	0.144	-0.11(-0.74,0.51)	0.721	0.82(0.14,1.49)	0.017
Physically active	0.18(-0.04,0.39)	0.083	1.00(0.40,1.60)	0.001	0.84(0.21,1.48)	0.009
Alcohol use	-0.14(-0.27, -0.01)	0.020	-1.65(-2.94, -0.36)	0.012	-1.78(-3.1,-0.47)	0.008
Current smoking	-0.04(-0.14,0.06)	0.317	-0.81(-2.38,0.76)	0.310	-0.22(-1.65,1.21)	0.759
Clinical characteristics [Mean]						
Weight (Kg)	-0.45(-1.22,0.32)	0.241	-0.97(-1.79, -0.16)	0.019	-0.39(-1.41,0.63)	0.449
Waist circumference (cm)	-4.1(-6.13, -2.07)	0.000	-2.16(-4.35,0.03)	0.054	-1.91(-3.67,-0.15)	0.034
Waist-to-hip ratio	-0.05(-0.08, -0.02)	0.004	-0.02(-0.04,0)	0.080	-0.02(-0.04,0)	0.014
Systolic BP (mmHg)	-1.67(-4.34,0.99)	0.214	-2.47(-6.11,1.18)	0.184	-0.82(-4.66,3.01)	0.673
IDRS Score	-9.4(-12.62,-6.18)	0.000	-2.69(-5.85,0.46)	0.094	-1.42(-4.55,1.72)	0.374
Biochemical characteristics [Mean]						
FPG (mg/dl)	1.26(-1.15,3.67)	0.298	-2.72(-5.71,0.28)	0.075	-1.23(-5.65,3.2)	0.587
2-hr plasma glucose(mg/dl)	-2.42(-9.9,5.06)	0.518	-13.84(-23.72,-3.95)	0.006	-6.64(-17.31,4.03)	0.222
HbA1c (percent)	-0.07(-0.21,0.07)	0.335	-0.1(-0.22,0.02)	0.096	-0.11(-0.24,0.01)	0.073
LDL cholesterol (mg/dl)	-1.6(-8.72,5.52)	0.654	-4.71(-12.14,2.71)	0.213	-4.27(-11.84,3.31)	0.269
Health-related quality of life*[Mean]						
Physical component summary	3.18(1.23,5.13)	0.002	2.36(0.21,4.51)	0.031	0.96(-1.24,3.16)	0.391
Mental component summary	2.9(0.27,5.53)	0.031	2.15(-0.16,4.45)	0.068	1.93(-0.22,4.08)	0.079
SF-6D	0.09(0.06,0.13)	0.000	0.06(0.02,0.1)	0.004	0.04(0,0.08)	0.058

*Higher scores show better quality of life; FPG = Fasting Plasma Glucose; PL, peer leader

Note: We used McNemar’s Chi-square test (for categorical variables) and Paired t-test (for continuous variables) compute differences between baseline and 12 months among peer leaders. We used and *logistic regression-based pairwise comparison to compute differences in predicted proportions and ANOVA with pairwise comparison to compute differences in marginal means between peer leaders and other participants at 12 months and 24 months. All models were adjusted for age and gender. Additionally, effects at 12 months and overall effects at 24 months were adjusted for baseline values of the respective variables. P values < 0.05 were considered to be statistically significant.*

## Discussion

In one of the first group-based, peer-led lifestyle interventions for diabetes prevention implemented in low- and middle-income countries, lay peer leaders showed improvements and greater health improvements compared to other program participants in health behaviors, anthropometric and clinical measures and quality of life.

Although chosen by their fellow group participants, selected peer leaders tended to be better educated as well as having healthier characteristics in terms of fruits and vegetable consumption, tobacco use, health related quality of life, systolic blood pressure and LDL cholesterol. These differences notwithstanding, they went on to show significant improvements during the 12 months of the K-DPP in alcohol use, waist circumference, waist-to-hip ratio, IDRS scores and all three measures of HRQoL. They largely sustained these changes to follow-up 24 months after baseline for alcohol, improvements in waist-to-hip ratio, the mental component of HRQoL and SF-6D. Changes among the peer leaders exceeded those among other participants for 5 of 15 variables at both 12 months and 24 months. Thus, peer leaders improved during the program, sustained several improvements, and showed a number of greater improvements and sustained improvements compared to other participants.

For peer leaders, most of the changes in health behaviours and outcomes that were observed at 12 months were maintained at 24 months, with no significant improvement or worsening between 12 months and 24 months. Besides, the changes in fruit and vegetable intake and physical component summary of HRQoL were extended to 24 months. However, the changes in waist circumference and IDRS were only partially maintained at 24 months.

The Kerala Diabetes Prevention Program has already been found to be both effective and cost-effective in reducing cardiometabolic risk among adults at high risk of diabetes over two years period [32]. This study demonstrated greater health benefits for peer leaders, as compared to other participants, on several health behaviour and outcome measures, including physical activity, fruit and vegetable intake, and alcohol consumption. In this regard, the findings of this study indicated that peer support programs could lead to greater improvements in health behaviors and outcomes among the peer leaders than other participants in improving common diabetes risk factors.

Previous studies have shown that training people at high-risk of diabetes on diabetes prevention can enable them effectively to organize and lead peer support groups for diabetes prevention [17, 21]. Our findings suggested that involvement in peer support programs for diabetes prevention as a peer leader can lead to greater health benefits, in addition to the communication and leadership skills that are usually developed during training and delivery of the intervention. Therefore, the findings of this study add an important dimension of greater health benefits of peer support interventions for the peer leaders relative to other participants, such as in fruit and vegetable intake and waist-to-hip ratio.

The peer leaders were selected among the participants by the participants. They were not recruited to be “models of health” to help their peers. Rather, they were recruited and had joined as individuals themselves at elevated risk of diabetes. Nevertheless, they were chosen to be peer leaders in the first meeting, suggesting some recognition of their value as role models or motivators by fellow participants and, perhaps, some putting themselves forward for this role. In this regard, it is noteworthy that the significant differences between peer leaders and other participants are primarily behavioral – smoking, diet, physical quality of life, whereas the two clinical/biochemical differences, systolic blook pressure and LDL were still within the normal or borderline range. Thus, the peer leaders may have acted and presented themselves as living a healthier lifestyle than others so that their greater benefits through the K-DPP may have reflected, in part, initial behavior and motivation.

The greater exposure of peer leaders to education content through the two rounds of peer leaders’ training sessions may have contributed to the greater benefits for peer leaders. Preparation for and assuming leadership role for peer support groups, as it promotes positive attitude and consolidates gained knowledge, may have also contributed to the greater benefits for peer leaders. A rewarding experience as a peer leaders could have also strengthened the achievement of behavioral goals among peer leaders [33, 34]. The modest baseline advantages may also have made peer leaders more likely to benefit from the *motivational theory of role modeling* [35]. Based on this theory, the responsibility of providing different forms of peer support services to other participants may motivate peer leaders to reframe their identity as role models thereby shifting the focus from self to others [36]. For peer leaders, this reframing may have been a natural “next step” from their somewhat healthier lifestyles and willingness to serve. This would further lead to the adoption of healthy behaviors, such as a healthy diet, physical activity, and reduction of alcohol. This responsibility will also put positive pressure on peer leaders to enhance their professional growth by building job skills and career goals. However, rather than treating the categories of peer leader and participant as fixed and intrinsically different, an area for future research would be to develop ways to promote among all participants a sense of modelling for each other so as to recruit more broadly the beneficial processes of the theory of role modeling.

Complementary to the theory of role modeling, the K-DPP strongly emphasized the community and neighborhood context of both individual change and peer support. In contrast to Western individualistic models of behavior change that, for example, emphasize the individual’s role as an active decision maker, individual goals, and self-regulation, K-DPP sought to address unhealthy lifestyles collectively such as by considering of implications of choices for families, household cooperation and response to changes in behavior, household decision-making and household efficacy, and setting lifestyle goals with participants’ family members, household. In addition to groups choosing their peer leaders, the program emphasizes varied group and community activities. Consistent with these emphases, for example, 75% of participants reported an average of 11 contacts with peer leaders *outside* group sessions. Peer leaders, then, were recruited to key roles in a community program, not just leaders of classes or group meetings. This may have added to their feelings of attachment to the program and its health goals [23].

Given the training, additional responsibilities, and time commitment of peer leaders, superiority (or at least non-inferiority) of their health outcomes is desirable. It can make them good role models for their peers. The evidence of added health benefits of peer support programs for peer leaders has the potential to encourage more people to volunteer to be peer leaders/supporters in chronic disease prevention and control programs. It could also motivate peer leaders to increase their engagement in peer support interventions, thereby helping to sustain participants’ involvement as well as the sustainability of the group as a whole. Both of these factors are important in the sustainability and scale-up of peer support programs in chronic disease prevention and control, especially in low- and middle-income countries.

The differences in health behaviours and outcomes between peer leaders and other participants could also be due to other factors such as background characteristics of the peer leaders, the training of peer leaders, and their higher level of participation in the group sessions than other participants. All the peer leaders attended the initial training and 85% of them attended the refresher training too. Hence, peer leaders received a “higher dose” intervention as compared to participants. The training emphasized not just knowledge but also skills in goal setting, planning, and monitoring goal progress. Peer leaders also received support from the project team and other peer leaders. Relative to other participants, peer leaders had a higher level of attendance at peer group sessions. Besides, peer leaders had a higher level of education, and this might have affected the level of risk and health outcomes. The combined effect of all these factors might have contributed to greater behavior and health outcomes among peer leaders. Some of the effects, lasting only during the active intervention period, suggesting that the enhanced social support might have been the most crucial component for the effect of the interventions.

There are some limitations associated with this study. First, the number of peer leaders as compared to the number of participants was small and this might have affected the statistical power of the analysis. Second, although we adjusted for key sociodemographic variables and the baseline status of the measures, there is a possibility of residual confounding in the comparison of health behavior and outcome measures between peer leaders and their peers as educational status, leadership qualities, organizing skills, teamwork quality, and experience of peer leaders were not accounted for. Finally, some of the measures of health behaviors and outcomes were self-reported and the possibility of social desirability bias cannot be ruled out.

## Conclusions and recommendations

We found that lay peer leaders, who were trained to lead group-based peer support intervention for diabetes prevention, had greater improvements in health behaviours and outcomes such as participation in leisure-time physical activity, waist-to-hip ratio, and mental health component of HRQoL during the intervention period, as compared to other participants. Further studies are needed to examine the long-term health effects of these benefits in terms of reducing the risk of diabetes and other chronic diseases.

## Data Availability

The datasets generated and/or analyzed during the current study are not publicly available due to due other analyses proceeding but are available from the corresponding author on reasonable request.
